# Subphenotyping of critical illness: where protocolized and personalized intensive care medicine meet

**DOI:** 10.5935/0103-507X.20220069-en

**Published:** 2022

**Authors:** Fernando José da Silva Ramos, Allan M. França, Jorge Ibraim Figueira Salluh

**Affiliations:** 1 Anesthesiology, Pain and Intensive Care Medicine Department, Universidade Federal de São Paulo - São Paulo (SP), Brazil.; 2 Postgraduate Program of Internal Medicine, Universidade Federal do Rio de Janeiro - Rio de Janeiro (RJ), Brazil.; 3 Instituto D’Or de Pesquisa e Ensino - Rio de Janeiro (RJ), Brazil.

## INTRODUCTION

In recent decades, successful quality improvement initiatives in critical care have been tested, and among the included principles were to “do no harm” (which means to prevent intensive care unit-acquired complications and to avoid overtreatment) and to provide early interventions for acute conditions (*i.e.*, antibiotics for sepsis, as well as reperfusions for stroke and myocardial infarction). However, a degree of imbalance is present in the abovementioned premises. Most of the improved outcomes that have been observed in critical care in the past decades can be attributed to the prevention of complications (*i.e.*, nosocomial infections, protective ventilation and deep vein thrombosis) and to the treatment of well-defined etiologic conditions (*i.e.*, stroke and myocardial infarction), thus resulting in very prevalent syndromes (*i.e.*, acute respiratory distress syndrome - ARDS and sepsis) comprising a minor portion of the effective treatments, which partially explains their current elevated mortality rates. Proponents of the protocolized care have used these arguments to promote the broad implementation of well-standardized, evidence-based practices aiming to reduce variations of care and to improve outcomes. Furthermore, those individuals proposing personalized care state that a physiology-based approach would hold the key to improving outcomes in patients with shock, acute respiratory failure (ARF), brain injury and other conditions.

Studies concerning psychology and decision-making show that when we evaluate and compare a range of data points, we tend to neglect the relative strength of the evidence and its spectrum and treat the evidence as being simply binary. This is known as the “binary bias”. Somehow, this approach (coupled with the tendency in critical care to group heterogeneous patient populations under syndromes (*i.e.,* ARF, ARDS, sepsis and *delirium*) is well represented in the treatment protocols that are available in intensive care units (*i.e.*, sepsis and ventilator-associated pneumonia bundles). In contrast, the pure physiology-based approach has been the basis of several failed interventions in ventilatory support, glucose control and *delirium,* among other interventions.

Lessons from other areas of medicine have shown that the integration of both initiatives is likely more effective. A good example comes from oncology, wherein the mapping of patient characteristics (such as functional capacity and genetic profiles), aspects of the current disease (such as tumor type, gene signature and extension of disease) and patient preferences will establish eligibility for a treatment protocol. This eligibility (when combined with the aforementioned characteristics) is translated into prognostic features and the potential of the treatment response.

In critical care, we still struggle to merge a personalized understanding of the patient with a wide choice of effective treatment protocols.

### Subphenotype-targeted therapies for critically ill

**Figure 1:**
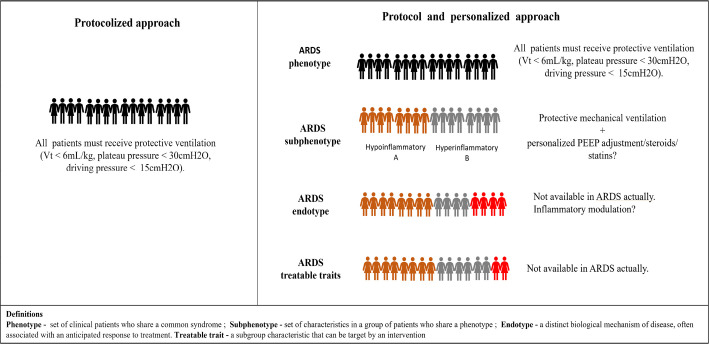
Example of protocolized versus protocolized and personalized approaches in the future.

Source: adapted from Reddy K, Sinha P, O’Kane CM, Gordon AC, Calfee CS, McAuley DF. Subphenotypes in critical care: translation into clinical practice. Lancet Respir Med. 2020;8(6):631-43.^(1)^

In recent decades, most trials and interventions in critical care have failed to improve relevant patient outcomes through pharmacological and mechanical ventilation strategies, as well as via hemodynamic resuscitations for heterogeneous and complex critical care illness (most often occurring in syndromic conditions). These trials are very helpful for demonstrating the potential iatrogenicity of a “one size fits all” approach intervention to syndromic conditions. However, they also showed that looking beyond the heterogeneous diagnoses may provide valuable insights into clinical characterization, clinical trial entry criteria and ultimate responsiveness to treatment. Advances in omics science (such as genomics, proteomics and metabolomics), analytic tools and big data have allowed us to identify novel disease subgroups (known as subphenotypes) that increased the biological and clinical understanding of features, outcomes and responses to treatment in prevalent and severe syndromes, such as sepsis, ARDS, *delirium*, acute kidney injury (AKI) and other disorders.^(1,2)^ Reddy et al. have recently proposed definitions for grouping patients by dividing by phenotype, subphenotype, endotype and treatable type (Figure 1).^(1)^ Such an approach may better inform outcomes and improve guidance for therapies.^(1,3)^

Vt - tidal volume; ARDS - acute respiratory distress syndrome; PEEP - positive end-expiratory pressure.

In sepsis, most of the randomized controlled trials focusing on pharmacological therapies have failed to improve outcomes. The Surviving Sepsis Campaign^(4)^ currently presents the best available evidence for sepsis care, but many criticisms have been made arguing that not all patients should have the same approach.^(5)^ Seymour et al. identified four clinical phenotypes of sepsis that correlated with host-response patterns and clinical outcomes.^(6)^ In this study, the authors used simulations of 3 large multicenter trials and estimated that the treatment benefit or harm was sensitive to phenotype distributions. When considering that almost completed clinical trials did not recognize heterogeneity in the treatment effects by using clinical phenotypes, further research is needed to determine the utility of these phenotypes in clinical care. For example, in a simulation analysis, the author found that early goal-directed therapy was beneficial for the “alpha phenotype” and harmful for the “delta phenotype”. Zhang et al. analyzed data from 14,993 patients and identified four subphenotypes of sepsis that demonstrated different mortality rates and responsiveness to fluid therapy.^(7)^ More recently, in a secondary analysis of multicenter registries in Japan, Kudo et al. recognized four sepsis phenotypes by using coagulopathy criteria and observed that in patients with severe organ dysfunction and coagulopathy, the use of thrombomodulin was associated with a lower mortality rate.^(8)^

In ARDS, numerous studies have increased our knowledge of pharmacotherapy and ventilation support; however, mortality rates have been stable in recent years, ranging from approximately 30% - 40%.^(9,10)^ Currently, the treatments associated with improved outcomes in ARDS include protective ventilatory strategies, prone therapy and the use of neuromuscular blockers, with the last two strategies being used in patients with moderate to severe ARDS.^(10)^ Thus, the currently effective interventions are mostly related to the prevention of ventilator-induced lung injury (which is a potentially iatrogenic factor and not a modulation or treatment of the disease and its underlying pathophysiologic features).

By approaching ARDS with the subphenotyping perspective, Calfee et al. identified two subphenotypes of ARDS patients (the hyperinflammatory and hypoinflammatory subphenotypes) with different prevalences, mortalities and responses to ventilatory strategy.^(11)^ Recently, Duggal et al. used nine clinical variables to analyze data from ARDS trials and identified 2 subphenotypes. Patients with subphenotype B showed increased levels of proinflammatory markers, higher mortality and a longer duration of ventilation than those patients with phenotype A.^(12)^ In addition, Calfee et al. found that outcomes vary with statin treatment according to ARDS phenotype, with better responses observed in patients with the “hyperinflammatory phenotype”.^(13)^

More recently, studies on coronavirus disease 2019 were also performed and could help to identify clinical and immunophenotypes associated with outcomes, as well as potentially identify responses to specific therapies.^(14)^

As summarized above, a better understanding of clinical and laboratory profiles associated with outcomes and responses to treatment (subphenotyping) may provide a way to transition from dichotomic protocols (where we will treat or not treat critically ill patients based on the presence of the diagnosis) to a more refined approach, wherein protocolized care would be widely provided to syndromic conditions (such as sepsis, ARDS, *delirium* or AKI) in a more personalized approach. Decision trees and algorithms can help clinicians in navigating through these protocols in a similar way to how oncologists apply their treatment choices. In this scenario of multiple possible treatment combinations for each patient with a given syndrome, protocols will ensure adherence to evidence-based medicine.

## CONCLUSION

Critically ill patients and critical care syndromes are complex. Protocolized care for the most common syndromes adds significant value because their use allows physicians and the multidisciplinary team to deliver the best evidence-based medicine with less variation. However, they currently demonstrate limited options and a “one size fits all” approach. Grouping patients into phenotypes, subphenotypes and endotypes will allow for better and tailored implementations of protocols in a more personalized way for critical care patients.
